# Over-expression of adenosine deaminase in mouse podocytes does not reverse puromycin aminonucleoside resistance

**DOI:** 10.1186/1471-2369-11-15

**Published:** 2010-07-22

**Authors:** Gaëlle Brideau, Alain Doucet

**Affiliations:** 1 Laboratoire de génomique, physiologie et physiopathologie rénales, Université Pierre et Marie Curie, Institut National de la Santé et de la Recherche Médicale, Centre National de Recherche Scientifique, 15 rue de l'Ecole de Médecine, Paris, 75270 cedex 6, France

## Abstract

**Background:**

Edema in nephrotic syndrome results from renal retention of sodium and alteration of the permeability properties of capillaries. Nephrotic syndrome induced by puromycin aminonucleoside (PAN) in rats reproduces the biological and clinical signs of the human disease, and has been widely used to identify the cellular mechanisms of sodium retention. Unfortunately, mice do not develop nephrotic syndrome in response to PAN, and we still lack a good mouse model of the disease in which the genetic tools necessary for further characterizing the pathophysiological pathway could be used. Mouse resistance to PAN has been attributed to a defect in glomerular adenosine deaminase (ADA), which metabolizes PAN. We therefore attempted to develop a mouse line sensitive to PAN through induction of normal adenosine metabolism in their podocytes.

**Methods:**

A mouse line expressing functional ADA under the control of the podocyte-specific podocin promoter was generated by transgenesis. The effect of PAN on urinary excretion of sodium and proteins was compared in rats and in mice over-expressing ADA and in littermates.

**Results:**

We confirmed that expression of ADA mRNAs was much lower in wild type mouse than in rat glomerulus. Transgenic mice expressed ADA specifically in the glomerulus, and their ADA activity was of the same order of magnitude as in rats. Nonetheless, ADA transgenic mice remained insensitive to PAN treatment in terms of both proteinuria and sodium retention.

**Conclusions:**

Along with previous results, this study shows that adenosine deaminase is necessary but not sufficient to confer PAN sensitivity to podocytes. ADA transgenic mice could be used as a background strain for further transgenesis.

## Background

Nephrotic syndrome is defined by abnormal urinary excretion of proteins leading to hypoalbuminemia. These biological signs are secondary to alterations of the glomerular filtration barrier. The primary forms of the disease correspond to either genetic alterations of proteins involved in the glomerular filtration barrier or to the idiopathic nephrotic syndrome which is due to a circulating factor that functionally alters the glomerular barrier. Whatever its etiology, nephrotic syndrome is always associated with renal retention of sodium which, along with alterations of the permeability properties of the capillary wall, promotes ascites and/or edema [1]. The most widely used animal model to study sodium retention in nephrotic syndrome is a rat model that reproduces the biological and clinical signs of the human disease [2,3]. It is induced by a single injection of puromycin aminonucleoside (PAN), an adenosine derivative used as an antibiotics and anti-proliferative drug that also induces nephrotic syndrome in humans.

Previous studies in nephrotic rats have shown that sodium retention results from stimulation of its reabsorption in the aldosterone sensitive distal nephron [4-6] and from resistance of the terminal collecting duct to the effect of atrial natriuretic peptide [7,8]. Increased sodium reabsorption originates in principal cells where both apical sodium entry via the epithelial sodium channel ENaC and basolateral exit via Na,K-ATPase are increased [6,9]. Although ENaC and Na,K-ATPase are known targets of aldosterone [10,11], and although plasma aldosterone is increased in PAN nephrotic rats [3], sodium retention is independent of aldosterone [6,12]. As a matter of fact, sodium retention is not accounted for by variations in any endocrine system since it affects only the treated kidney in unilateral PAN-induced nephrotic syndrome [5]. Several alternate hypotheses have been proposed to explain sodium retention, but they have not been unambiguously validated, as this would require genetic invalidation of hypothesized target genes. Unfortunately mice do not develop nephrotic syndrome in response to PAN, and no mouse model of nephrotic syndrome featuring the signs of the human disease exists yet.

Interspecies differences in sensitivity to PAN have been associated with differences in the adenosine metabolism pathway. Thus, Nosaka et al. [13] showed that sensitivity to PAN of different species was correlated with their renal adenosine deaminase (ADA) activity: Among the species they studied, rats and mice displayed the highest and lowest ADA activity respectively, and were the two prototypes of PAN sensitivity and resistance in term of proteinuria. The same group also showed that pre-treatment of rats with the ADA inhibitor 2'-deoxycoformycin prevented PAN-induced proteinuria and glomerular lesions [13]. More recently, Xia et al. [14] showed that adenosine and PAN enter cells via the plasma membrane amine transporter (PMAT) which is expressed specifically in podocytes in both rat and human. Overexpression of PMAT in MDCK cells increased their sensitivity to PAN, an effect which was abolished by decynium 22, a potent inhibitor of PMAT [14]. Thus, low expression of PMAT in mouse podocyte might also account for PAN resistance.

In an attempt to develop a mouse line sensitive to PAN through induction of normal adenosine metabolism in podocytes, we sought to determine whether or not PMAT expression in mouse glomerulus might be limiting in order to engineer mice expressing ADA and, if necessary PMAT, in their podocytes through transgenesis using a podocyte specific promoter. The sensitivity to PAN was evaluated on the basis of its ability to induce urinary excretion of proteins and to reduce sodium excretion in transgenic mice as compared to littermates. Rats were studied in parallel as positive controls.

## Methods

### Transgenesis plasmid construction

**Figure 1:**
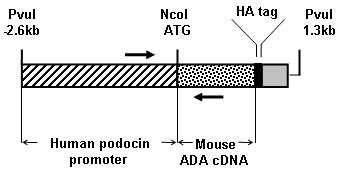
**Schematic structure of the NPHS2-promoter/ADA transgene**. The transgene construct carried a 2.6-kb DNA fragment located upstream the translated region of the human NPHS2 (hatched) gene followed by the mouse ADA cDNA (dotted). A HA-tag was introduced at the 3' end of ADA (black), just before the stop codon. Finally, the poly-adenylation (grey) sequence corresponds to the SV40 Late poly(A) from pCi vector. Arrows indicate the location of primers used for PCR genotyping of mice.

Plasmid containing the human NPHS2 promoter was kindly provided by C Antignac (Hôpital Necker, Paris). Oligonucleotide primers were designed to PCR amplify a 2.6 kb fragment of NPHS2 promoter located upstream the NPHS2 initiation codon (sense: 5'-GAAGATCTCAGCTGGCCCTCCTATTTAGTCTCTCTGCCACC-3' and antisense: 5'-CCCGGCAGCTCTGACCATGGTACCCC-3'). The sense and antisense primers contained *Bgl*II-*Pvu*I and *Kpn*I sites respectively. This promoter fragment was cloned at the *Bgl*II and *Kpn*I sites of pCi vector (Promega) in place of CMVI.E promoter and intron sequences. Similary, oligonucleotide primers were designed to PCR amplify from mouse kidney cDNAs the full length mouse ADA cDNA (sense: 5'-AAGGAAAAAAGCGGCCGCGGAACCATGGCCCAGACACCCGCATTCAACAA-3', and antisense: 5'-TTCTCCTTTTGCGGCCGCCTAAGCATAATCCGGTACATCATACGGGTATTGGTATTCTCTGTAGAGCCGT-3'). These two primers contained a *Not*I site, and the antisense primer also encoded a hemagglutinin tag before the stop codon. The ADA cDNA was cloned at *Not*I site of pCi vector, dowstream the NPHS2 promoter fragment. Finally, the modified pCi vector was digested with *Nco*I to remove the vector fragment remaining between the promoter and the ADA cDNA. The sequences of all constructs were checked by sequencing. After amplification and *Pvu*I digestion, the transgenesis plasmid (Figure 1) was purified. Generation of transgenetic mice in FVB/n genetic background was performed by the Service d'Expérimentation Animale de Transgénèse (CNRS UPS 44, Villejuif, France). Generation of this mouse transgenic line and its study were performed with the approval of the French Ministère de l'Enseignement Supérieur et de la Recherche (approval # 5197, april 16, 2009).

### Transgene detection by PCR

Transgenic mice were identified by PCR on DNA recovered from tail biopsies. Tail fragments of 1 month-old mice were lyzed overnight at 55°C in TNES solution (100 mM Tris-HCl pH 8.5, 200 mM NaCl, 10 mM EDTA, 0.2% SDS) containing 0.66 mg/ml proteinase K (Invitrogen). Proteinase K was inactivated 5 min at 95°C. The transgene was identified by PCR (GoTaq^® ^DNA polymerase, Promega, 35 cycles, Tm: 65°C) using primers located in the promoter and in ADA regions respectively (Figure 1): sense, 5'-TGCAGACACGCACTTTTCAA-3'; reverse, 5'-GCCATCAAGAGGATCGCCTA-3'

### Microdissection

Glomerulus, cortical collecting ducts (CCD) and proximal convoluted tubules (PCT) were dissected from liberase-treated kidneys (Blendzyme 2, Roche Diagnostics) as previously described [15]. Briefly, the left kidney was perfused *in situ *with 6 ml of Hank's solution supplemented with 1 mM glutamine, 1 mM pyruvate, 0.5 mM MgCl_2_, 0.1% bovine serum albumin, 20 mM Hepes, and 0.015% liberase (w/v), pH 7.4. Thin pyramids were cut from the kidney and incubated in 0.006% liberase solution for 20-25 min at 30°C and thoroughly rinsed in microdissection solution supplemented with a cocktail of protease inhibitors (Complete EDTA-free, Roche Diagnostics) when used for western blot. For RNA extraction, tissues were isolated under Rnase-free conditions.

### Western blot analysis

After microdissection, pools of 50 to 60 glomerulus, PCT or CCD were solubilized at 95°C for 5 min after addition of 2× Laemmli. SDS-PAGE was performed on 10% polyacrylamide gels and proteins were transferred to Hybond™-P membrane (GE Healthcare) using standard procedure. Blots were blocked in 5% non-fat dry milk in TBS-Nonidet P-40 buffer (50 mM Tris base, 150 mM NaCl, 0.2% Nonidet P-40) and incubated with a specific rat anti-HA antibody (Roche diagnostics; dilution 1/1 500), and incubated with a secondary horseradish peroxidase-linked anti-rat antibody (Jackson Immunoresearch, Ltd). Immunodetection was performed using enhanced chemiluminescence light detecting kit (Amersham, Arlinghton Heights, IL, USA).

### RNA extraction and RT-QPCR

RNAs were extracted from pools of 50-60 glomerulus using RNeasy Plus Micro Kit (Qiagen) according to the manufacturer's instructions. Reverse transcription was performed on 80% of glomerular RNA extract using the first-strand cDNA synthesis kit for reverse transcription-PCR (Roche Diagnostics), according to the manufacturer's instructions. The remaining 20% of glomerulus RNA extract were processed in parallel in the absence of reverse transcriptase and served as controls. No amplification product was detected in these controls in any experiment.

Real-time PCR was performed on a LightCycler (Roche Diagnostics) with the LightCycler 480 SYBR Green I Master qPCR kit (Roche Diagnostics) according to the manufacturer's instructions, except that the reaction volume was reduced to 10 μl. PCR was performed in the presence of cDNA corresponding to 1 glomerulus. Specific primers (available upon request) were designed using Light Cycler probe design software II (Roche Diagnostics).

### Animals

All animal experiments were carried out according the French legislation, under the responsibility of an enabled experimenter (AD, license #75-699 renewal). Nephrosis was induced in male Sprague Dawley rats (160-200 g, Charles River, France) by a single administration of PAN (150 mg/kg body wt, jugular vein). In mice, PAN was administered either once or twice (150-300 mg/kg body wt, retro-orbital or jugular vein injection).

Animals were acclimatized to metabolic cages for at least two days before PAN administration. 24-h urine was collected for determination of sodium, creatinine, and proteins on an automatic analyzer (Konelab 20i, Thermo, Cergy Pontoise, France). Urinary sodium and protein excretions were calculated as a function of urinary creatinine excretion. Animals were sacrificed at the end of the experimental period (6-10 days after PAN injection).

### Determination of ADA specific activity

The method used was previously described by Chinsky et al. [16]. Rat and mouse kidneys were placed in ice cold 0.1 M potassium phosphate, pH 7.4 buffer containing 1 mM 2-mercaptoethanol and were lysed by Polytron. Cell debris were removed by microfuge centrifugation (10 min at 4°C). Protein content of homogenates was estimated by the method of Bradford. ADA activity was assayed at room temperature in a reaction mixture containing 0.14 mM adenosine, 50 mM potassium phosphate, pH 7.4 and tissue homogenate. The decrease in absorbance at 260 nm resulting from deamination of adenosine to inosine was monitored every 10s with a spectrophotometer and the rate of inosine production was determined under initial rate conditions [17].

## Results

### Expression of ADA and PMAT in mouse and rat glomerulus

The expression levels of ADA and PMAT determined by RT-QPCR in mouse and rat glomerulus were compared. For this purpose, we calculated the amount of each transcript as:

in which K is a transcript-independent constant, L is the amplicon length, Eff is the PCR efficiency and C_p _is the threshold of fluorescence detection, determined as the number of PCR cycles at which the reaction fluorescence reaches its second derivative maximum [18].

**Figure 2:**
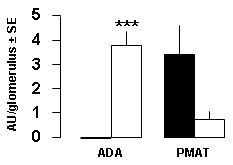
**Expression of PMAT and ADA mRNAs in rat and mouse glomerulus**. Expression of mRNAs was quantitated by QPCR on retro-transcribed mRNAs from microdissected mouse (black columns) and rat (open columns) glomerulus. Values refer to one glomerulus in either mouse or rat and are means ± SE from 5 mice and 6 rats. Statistical significance between groups was assessed by unpaired Student's t test: ***, p < 0.001.

Expression of ADA was very low in the glomerulus of mouse as compared to that of rat. In contrast, mRNA expression level of PMAT was ~4-fold higher in mouse glomerulus than in rat glomerulus, but the difference was not statistically significant (Figure 2). Thus, as opposed to ADA, PMAT did not appear to be a limiting factor for PAN metabolism by mouse podocytes. We therefore decided to promote normal adenosine metabolism in mouse glomerulus by generating a mouse line over-expressing ADA in podocytes.

### Transgenic mice

**Figure 3:**
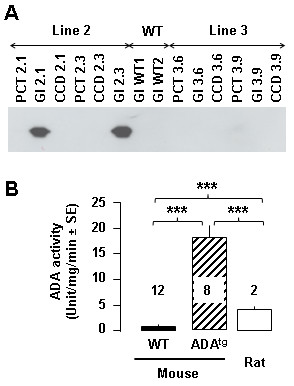
**Characterization of ADA transgenic mice**. A. Western blot analysis of ADA expression. Expression of ADA was analyzed using an anti-HA antibody in glomerulus (Gl), proximal convoluted tubule (PCT) and cortical collecting duct (CCD) from two mice from lines 2 (2.1 and 2.3) and 3 (3.6 and 3.9). Glomerulus from two wild type mice (WT) served as negative controls. Only mice from line 2 expressed ADA, and expression was restricted to the glomerulus. B. ADA activity in mouse and rat kidney. ADA activity was determined in kidney homogenates from wild type mice, ADA^tg ^mice and control rats. Values (units/mg tissue protein/min) are means ± SE from several samples (number indicated above columns). Statistical differences between groups were assessed by variance analysis: ***, p < 0.001.

For this purpose, a 2.6-kb fragment of DNA located upstream from the human NPHS2 transcribed region was selected to drive the expression of ADA in podocytes. This fragment was selected because its gene product, podocin, is specifically expressed in podocytes [19] and because it was previously used successfully to drive expression of another transgene in these cells [20]. Trangenic mice were generated by pronuclear injection in FVB/n mice. Five transgenic founders were identified by PCR analysis of genomic DNA, among which one died a few weeks after birth, one was unable to reproduce, and one failed to transmit the transgene to its progeny. The last two founders transmitted the transgene to their progeny (mouse lines 2 and 3) but only line 2 expressed ADA at the protein level (Figure 3A). As expected from the promoter used to drive ADA expression, kidney expression was restricted to the glomerulus.

Because introduction of the HA-tag could have altered protein function, we evaluated ADA activity in the kidney of transgenic mice from line 2 (ADA^tg^). ADA activity was ~18-fold higher in ADA^tg ^mice than in wild types (Figure 3B). It was also ~4-fold higher than in rat kidney.

### Effect of PAN

**Figure 4:**
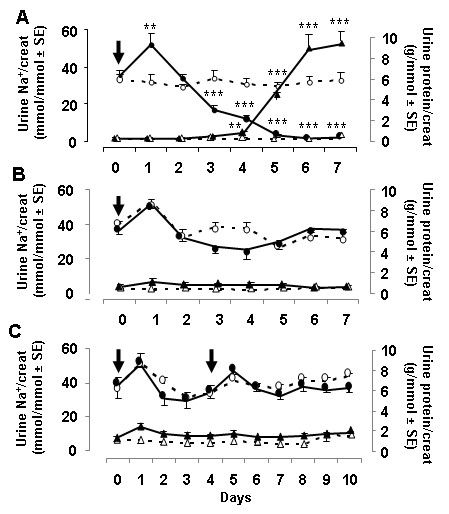
**Effect of PAN treatment on urinary excretion of proteins and sodium**. A. Effect of PAN in rats. PAN-treated (150 mg/kg body wt at day 0, solid lines) and -untreated rats (dotted lines) were housed in metabolic cages and their daily excretion of sodium (circles) and proteins (triangles) was monitored for 7 days. Results, expressed as a function of creatinine excretion, are means ± SE from 6 rats in each group. Statistical significance between treated and untreated rats was assessed by unpaired Student's t test: **, p < 0,025; ***, p < 0,001. B and C. Effect of PAN in wild type and ADA^tg ^mice. Wild type (dotted lines) and ADA^tg ^mice (solid lines) received a single (B) or two (C) injections of PAN (150 mg/kg body wt at day 0 and day 4, arrows) and were monitored for 7-10 days for urinary excretion of sodium (circles) and proteins (triangles). Results, expressed as a function of creatinine excretion, are means ± SE from 6 mice in each group except the ADA^tg ^group treated once (n = 9). Values statistically different from controls (day 0) were assessed by variance analysis followed by Bonferonni test: *, p < 0.05.

As previously reported [3,9], a single injection of PAN (150 mg/kg body wt) to rats induced a marked nephrotic syndrome with severe proteinuria (~10 g/mmol creatinine) and decreased sodium excretion (< 5 mmol/mmol creatinine) (Figure 4A). Note that administration of PAN induced a transient (day 1) increase in sodium excretion which has been attributed to cytotoxicity. In contrast, a single administration of PAN (150 mg/kg body wt) to wild type mice did not alter significantly their urinary excretion of proteins and sodium, except for the transient increase in sodium excretion at day one (Figure 4B, dotted lines). Note also that conversely to rats, mice displayed a basal proteinuria, in the range of 0.5-2 g/mmol creatinine. In ADA^tg ^mice, PAN neither increased proteinuria nor induced marked sodium retention. However, it slightly and transiently reduced sodium excretion, as compared with wild type mice, at days 3 and 4 (Figure 4B, solid lines). Extending the monitoring up to 14 days failed to reveal proteinuria or sodium retention in either wild type or ADA^tg ^mice (data not shown). Higher doses of PAN (up to 300 mg/kg body wt) also failed to induce a nephrotic syndrome in either group of mice (data not shown)

In a final experimental series, we investigated whether a second administration of PAN might amplify its transient effect on sodium excretion and induce proteinuria in ADA^tg ^mice. Figure 4C shows that two successive administrations of PAN (150 mg/kg body wt) at days 0 and 4 failed to induce proteinuria or sodium retention. In contrast, the two injections promoted a transient increase in sodium excretion, attesting the efficiency of PAN.

## Discussion

**Figure 5:**
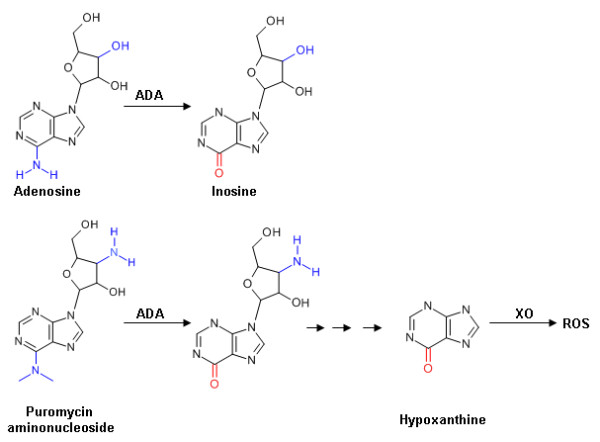
**Structure of adenosine, PAN and metabolites**. Blue and red lettering indicate the structural differences between adenosine and PAN, and the changes induced by ADA action respectively. ADA, adenosine deaminase; XO, xanthine oxidase; ROS, reactive oxygen species.

PAN-induced nephrotic syndrome in rat has been widely used for determining the structural alterations of the filtration barrier responsible for proteinuria and the functional changes responsible for tubular sodium retention. However, the mechanism of action of PAN is not fully understood. PAN is thought to promote proteinuria through a cytotoxic effect on podocytes. Early experiments in which a unilateral nephrotic syndrome was induced by transient infusion of PAN in one kidney while preventing its access to the general circulation have demonstrated that proteinuria results from a rapid and direct effect of PAN on the kidney. Several observations suggest that PAN needs to be metabolized to be nephrotoxic. Firstly, inhibition of ADA prevents PAN-induced proteinuria in rats [13]. As a matter of fact, PAN is a structural analogue of adenosine, the natural substrate of ADA (figure 5). However, PAN differs from adenosine by an amino group substitution of the hydroxyl group on carbon 3 of the ribose ring, and by a dimethylamino substitution of the purine amino group. Thus, it is unclear whether ADA catalyzes the deamination of the ribose or the removal of the dimethylamino residue of the purine, or both. Secondly, PAN-induced nephrotic syndrome is associated with the early production of reactive oxygen species (ROS) in the glomerulus [21-23] and treatment with ROS scavengers or antioxidants reduces or prevents proteinuria [22,24,25]. Interestingly, hypoxanthine is a metabolite of PAN [26] generated by the removal of the dimethylamino residue of the purine that acts as a substrate for ROS generation by xanthine oxidase [24].

Because the resistance of mouse podocytes to PAN was attributed to their deficiency in adenosine deamination pathway [13], we attempted to render mouse podocytes sensitive to PAN by promoting the expression of the adenosine deamination pathway that has been observed in rat podocytes. In order to be metabolized by ADA, PAN first has to enter podocytes via PMAT [14]. In this study we showed that PMAT is expressed in mouse glomerulus at least at the same level as in rat glomerulus, and through transgenesis we promoted a podocyte ADA activity slightly higher than in rat. Transgenesis was performed on FVB mice because this background has proven to be more susceptible to the development of proteinuria and ascites [27,28]. Nonetheless, whatever the dose and/or the number of PAN injections, these ADA transgenic mice developed neither proteinuria nor sodium retention. These results suggest that PAN metabolism by ADA is not sufficient to induce PAN sensitivity in mouse.

Over the past decade, many laboratories tried to develop a mouse model mimicking human nephrotic syndrome (Table 1). Three different strategies were developed. The first one consisted in selecting or generating a genetic background to render mice sensitive to PAN, as attempted in this study. The second one was to induce proteinuria by altering the genetic expression of proteins central to the filtration barrier. The last one aimed at generating idiopathic nephrotic syndrome through induction of the production of a permeability factor by immune cells.

**Table 1:** Animal models of proteinuria

Models	Proteinuria	Na handling	Ref
Injection of PAN			
Sprague Dawley rat	10 g/mmol creat	Ascites, edema	[9]
	90 mg/mg creat	Ascites, edema	[9]
	3 g/24 h/kg body wt	Ascites, edema	[9]
	30 mg/ml	Ascites, edema	[9]
Wild type B6/D2 mouse	0.03 mg/mg creat		[29]
ApoE^-/- ^mouse	0.2 mg/ml		[30]
Cox2 tg mouse	0.07 mg/mg creat		[29]
PRO/Re mouse	0.7 g/24 h/kg body wt		[31]

Injection of adriamycin			
Sprague Dawley rat	25 mg/mg creat	Na retention	[9]
B6/D2 mouse	0.04 mg/mg creat		[41]
Balb/c mouse	23 mg/mg creat		[42]
Balb/c mouse	226 mg/mg creat		[43]
129 mouse	120 mg/mg creat	Ascites	[44]

Genetically modified mice			
Laminin α5^-/-^	200 mg/mg creat		[32]
Laminin β2^-/-^	12 mg/ml		[33]
α-actinin-4^-/-^	+ +		[34]
Nphs2^-/-^	+ + +	HTA	[36]
CD2-AP^-/-^	+ + +		[35]
CD151^-/-^	+ + +	Edema	[27]

Human CD34^+ ^engraftment			
NOD/SCID mouse	0.04 mg/mg creat		[37]

Proteinuria in PAN nephrotic rats is given as a function of the different units found in the literature. + + and + + + refers to increases in proteinuria as determined by western blotting. When available, functional data related to renal sodium handling are provided; HTA, hypertension.

Results from the present study as well as several previous ones [13,29] have documented the resistance of mouse to the nephrosis-inducing effect of PAN. Increased sensitivity to PAN, in term of proteinuria, has been reported in mice over expressing cycloxygenase-2 in a podocyte-specific manner (nephrin promoter) [29] as well as in apolipoprotein E deficient, hypercholesterolemic mice [30]. However, in both cases, proteinuria was moderate (3- to 5-fold increase) and was rapidly reversible as it was no longer detected after 8 days. In contrast, Kanwar et al. reported over 30 years ago that repeated administration of PAN induced a massive, nephrotic-range proteinuria in hyperprolinemic mice (PRO/Re) with a natural deficiency in proline oxidase [31]. The possible link between PAN metabolism and proline oxidase remains obscure and, curiously, these results have never been confirmed since their publication, despite the major interest in disposing of a mouse model of nephrotic syndrome. No information is available regarding sodium metabolism and/or edema or blood pressure in any of these models.

To alter the glomerular filtration barrier, investigators have targeted genes encoding proteins essential for the structure and function of either the glomerular basement membrane (laminin α5 and β2) or the podocytes and slit diaphragm (Nphs2, CD2-AP, CD151 and α-actinin-4). Genetic inactivation of laminin α5 induced a marked proteinuria in some but not all mice [32]. Laminin β2-deficient mice also displayed marked proteinuria, but the mice did not grow normally and died prematurely [33]. A similar phenotype was developed by α-actinin-4- or CD2-associated protein-deficient mice [34,35]. These results clearly demonstrate the role of the targeted genes in the filtration barrier, but prove that the transgenic mouse lines generated are poor models for studying the mechanism of sodium retention, mainly because there is no evidence that these mice develop edema and/or ascites. This is possibly due to the premature death of all these gene-targeted mice. To circumvent the growth deficit associated with proteinuria during the development period, a model of inducible inactivation of podocin based on Cre-loxP technology was recently reported [36]. Interestingly, upon inactivation of podocin, these mice showed proteinuria, although it was not quantified, and hypertension, but no ascites or edema was described. In this model also, mice died prematurely as the median survival was 11 weeks following podocin loss. Mice with inactivation of CD151, a tetraspanin involved in the adhesion of podocytes to the basement membrane as well as in basement membrane integrity, deserve a special attention. As a matter of fact, CD151-deficient mice displayed marked, although not quantified, albuminuria and survived for at least 5 months. In addition, at this stage they were reported to exhibit edema [27]. Unfortunately renal function data (as opposed to morphological data) concerning these mice are scarce.

The last strategy consisted in developing a humanized model of nephrotic syndrome through engraftment of CD34 ^+ ^cells from nephrotic patients in NOD/SCID mice [37]. These mice developed mild proteinuria (3-fold increase) and the model is too tedious to be used routinely.

Adriamycin is also known to induce nephrotic syndrome in rat [38], and is often used to do the same in mouse. In rats, adriamycin induces a clear-cut proteinuria within 1-2 weeks after injection, although to a lesser extent than PAN. However, in terms of sodium retention the effect of adriamycin is transient and shows inter-individual differences in time-course [9], making it a poor model to study this phenomenon. In mouse, adriamycin-induced proteinuria is strain-dependent, with most strains being quite resistant to adriamycin and few ones developing nephrotic-range proteinuria within 1 week [39,40]. In an elegant study, Zheng et al. used inter-strain differences to identify by genome-wide analysis of linkage a locus on chromosome 16 associated with susceptibility to adriamycin, but they failed to identify the responsible gene [40]. They also found a modifier locus on chromosome 9. However, to our knowledge, sodium retention has not been documented in adriamycin-treated mice. Regarding tubular functions, a limit of the adriamycin model is that sensitive mouse strains develop not only focal segmental glomerulosclerosis but also tubulo-interstitial fibrosis and tubular lesions that rapidly progress towards renal insufficiency [40]. Thus, this is clearly not a relevant model to study increased sodium reabsorption.

## Conclusions

Enhancing adenosine deaminase activity in podocytes is not sufficient to render mice sensitive to PAN in terms of nephrotic syndrome. The Ada^tg ^mouse line that we have generated might be useful as background for future attempts to transfer PAN-sensitivity in mice, since adenosine deaminase activity remains necessary, but not sufficient, to provide sensitivity to PAN.

## Competing interests

The authors declare that they have no competing interests.

## Authors' contributions

GB designed the study, carried out the experiments, undertook data analysis and wrote the manuscript. AD designed the study and wrote the manuscript

## Pre-publication history

The pre-publication history for this paper can be accessed here:

http://www.biomedcentral.com/1471-2369/11/15/prepub

## References

[B1] Doucet A Favre G Deschenes G Molecular mechanism of edema formation in nephrotic syndrome: therapeutic implications Pediatr Nephrol 2007 22 12 1983 1990 10.1007/s00467-007-0521-3 17554565 PMC2064946

[B2] Frenk S Antonowicz I Craig JM Metcoff J Experimental nephrotic syndrome induced in rats by aminonucleoside; renal lesions and body electrolyte composition Proc Soc Exp Biol Med 1955 89 3 424 427 13254783 10.3181/00379727-89-21833

[B3] Pedraza-Chaverri J Cruz C Ibarra-Rubio ME Chavez MT Calleja C Tapia E del Carmen Uribe M Romero L Pena JC Pathophysiology of experimental nephrotic syndrome induced by puromycin aminonucleoside in rats. I. The role of proteinuria, hypoproteinemia, and renin-angiotensin-aldosterone system on sodium retention Rev Invest Clin 1990 42 1 29 38 2236972

[B4] Deschenes G Wittner M Stefano A Jounier S Doucet A Collecting duct is a site of sodium retention in PAN nephrosis: a rationale for amiloride therapy J Am Soc Nephrol 2001 12 3 598 601 11181809 10.1681/ASN.V123598

[B5] Ichikawa I Rennke HG Hoyer JR Badr KF Schor N Troy JL Lechene CP Brenner BM Role for intrarenal mechanisms in the impaired salt excretion of experimental nephrotic syndrome J Clin Invest 1983 71 1 91 103 10.1172/JCI110756 6848563 PMC436841

[B6] Lourdel S Loffing J Favre G Paulais M Nissant A Fakitsas P Creminon C Feraille E Verrey F Teulon J Hyperaldosteronemia and activation of the epithelial sodium channel are not required for sodium retention in puromycin-induced nephrosis J Am Soc Nephrol 2005 16 12 3642 3650 10.1681/ASN.2005040363 16267158

[B7] Perico N Delaini F Lupini C Benigni A Galbusera M Boccardo P Remuzzi G Blunted excretory response to atrial natriuretic peptide in experimental nephrosis Kidney Int 1989 36 1 57 64 10.1038/ki.1989.161 2554049

[B8] Rabelink AJ Koomans HA Gaillard CA Dorhout Mees EJ Renal response to atrial natriuretic peptide in nephrotic syndrome Nephrol Dial Transplant 1987 2 6 510 514 2964567

[B9] Deschenes G Doucet A Collecting duct (Na+/K+)-ATPase activity is correlated with urinary sodium excretion in rat nephrotic syndromes J Am Soc Nephrol 2000 11 4 604 615 10752519 10.1681/ASN.V114604

[B10] Barlet-Bas C Khadouri C Marsy S Doucet A Sodium-independent in vitro induction of Na+,K+-ATPase by aldosterone in renal target cells: permissive effect of triiodothyronine Proc Natl Acad Sci USA 1988 85 5 1707 1711 10.1073/pnas.85.5.1707 2830627 PMC279844

[B11] Masilamani S Kim GH Mitchell C Wade JB Knepper MA Aldosterone-mediated regulation of ENaC alpha, beta, and gamma subunit proteins in rat kidney J Clin Invest 1999 104 7 R19 23 10.1172/JCI7840 10510339 PMC408561

[B12] de Seigneux S Kim SW Hemmingsen SC Frokiaer J Nielsen S Increased expression but not targeting of ENaC in adrenalectomized rats with PAN-induced nephrotic syndrome Am J Physiol Renal Physiol 2006 291 1 F208 217 10.1152/ajprenal.00399.2005 16403831

[B13] Nosaka K Takahashi T Nishi T Imaki H Suzuki T Suzuki K Kurokawa K Endou H An adenosine deaminase inhibitor prevents puromycin aminonucleoside nephrotoxicity Free Radic Biol Med 1997 22 4 597 605 10.1016/S0891-5849(96)00349-8 9013123

[B14] Xia L Zhou M Kalhorn TF Ho HT Wang J Podocyte-specific expression of organic cation transporter PMAT: implication in puromycin aminonucleoside nephrotoxicity Am J Physiol Renal Physiol 2009 296 6 F1307 1313 10.1152/ajprenal.00046.2009 19357181 PMC2692440

[B15] Morla L Crambert G Mordasini D Favre G Doucet A Imbert-Teboul M Proteinase-activated receptor 2 stimulates Na,K-ATPase and sodium reabsorption in native kidney epithelium J Biol Chem 2008 283 42 28020 28028 10.1074/jbc.M804399200 18678869 PMC2661380

[B16] Chinsky JM Ramamurthy V Fanslow WC Ingolia DE Blackburn MR Shaffer KT Higley HR Trentin JJ Rudolph FB Knudsen TB Developmental expression of adenosine deaminase in the upper alimentary tract of mice Differentiation 1990 42 3 172 183 10.1111/j.1432-0436.1990.tb00759.x 2187728

[B17] Barton R Martiniuk F Hirschhorn R Goldschneider I The distribution of adenosine deaminase among lymphocyte populations in the rat J Immunol 1979 122 1 216 220 310828

[B18] Disset A Cheval L Soutourina O Duong Van Huyen JP Li G Genin C Tostain J Loupy A Doucet A Rajerison R Tissue compartment analysis for biomarker discovery by gene expression profiling PLoS One 2009 4 11 e7779 10.1371/journal.pone.0007779 19901995 PMC2771357

[B19] Chabardes-Garonne D Mejean A Aude JC Cheval L Di Stefano A Gaillard MC Imbert-Teboul M Wittner M Balian C Anthouard V A panoramic view of gene expression in the human kidney Proc Natl Acad Sci USA 2003 100 23 13710 13715 10.1073/pnas.2234604100 14595018 PMC263878

[B20] Macary G Rossert J Bruneval P Mandet C Belair MF Houillier P Van Huyen JP Transgenic mice expressing nitroreductase gene under the control of the podocin promoter: a new murine model of inductible glomerular injury Virchows Arch 456 3 325 337 10.1007/s00428-009-0840-9 19806361

[B21] Beaman M Birtwistle R Howie AJ Michael J Adu D The role of superoxide anion and hydrogen peroxide in glomerular injury induced by puromycin aminonucleoside in rats Clin Sci (Lond) 1987 73 3 329 332 2820648 10.1042/cs0730329

[B22] Gwinner W Landmesser U Brandes RP Kubat B Plasger J Eberhard O Koch KM Olbricht CJ Reactive oxygen species and antioxidant defense in puromycin aminonucleoside glomerulopathy J Am Soc Nephrol 1997 8 11 1722 1731 9355075 10.1681/ASN.V8111722

[B23] Rincon J Romero M Viera N Pedreanez A Mosquera J Increased oxidative stress and apoptosis in acute puromycin aminonucleoside nephrosis Int J Exp Pathol 2004 85 1 25 33 10.1111/j.0959-9673.2004.0368.x 15113391 PMC2517453

[B24] Diamond JR Bonventre JV Karnovsky MJ A role for oxygen free radicals in aminonucleoside nephrosis Kidney Int 1986 29 2 478 483 10.1038/ki.1986.24 3702206

[B25] Thakur V Walker PD Shah SV Evidence suggesting a role for hydroxyl radical in puromycin aminonucleoside-induced proteinuria Kidney Int 1988 34 4 494 499 10.1038/ki.1988.208 2848972

[B26] Nagasawa HT Swingle KF Alexander CS Metabolism of aminonucleoside-8-14C in the rat and guinea pig Biochem Pharmacol 1967 16 11 2211 2219 10.1016/0006-2952(67)90020-2 6076611

[B27] Baleato RM Guthrie PL Gubler MC Ashman LK Roselli S Deletion of CD151 results in a strain-dependent glomerular disease due to severe alterations of the glomerular basement membrane Am J Pathol 2008 173 4 927 937 10.2353/ajpath.2008.071149 18787104 PMC2543062

[B28] Maier SM Gross JK Hamlin KL Maier JL Workman JL Kim-Howard XR Schoeb TR Farris AD Proteinuria of nonautoimmune origin in wild-type FVB/NJ mice Comp Med 2007 57 3 255 266 17605340

[B29] Jo YI Cheng H Wang S Moeckel GW Harris RC Puromycin induces reversible proteinuric injury in transgenic mice expressing cyclooxygenase-2 in podocytes Nephron Exp Nephrol 2007 107 3 e87 94 10.1159/000108653 17890881

[B30] Cheng ZZ Patari A Aalto-Setala K Novikov D Schlondorff D Holthofer H Hypercholesterolemia is a prerequisite for puromycin inducible damage in mouse kidney Kidney Int 2003 63 1 107 112 10.1046/j.1523-1755.2003.00726.x 12472773

[B31] Kanwar YS Manaligod JR Krakower CA Aminonucleoside nephrosis in PRO/Re mice Proc Soc Exp Biol Med 1977 155 3 339 345 877132 10.3181/00379727-155-39802

[B32] Goldberg S Adair-Kirk TL Senior RM Miner JH Maintenance of Glomerular Filtration Barrier Integrity Requires Laminin {alpha}5 J Am Soc Nephrol 10.1681/ASN.2009091004 PMC2844312 20150535

[B33] Noakes PG Miner JH Gautam M Cunningham JM Sanes JR Merlie JP The renal glomerulus of mice lacking s-laminin/laminin beta 2: nephrosis despite molecular compensation by laminin beta 1 Nat Genet 1995 10 4 400 406 10.1038/ng0895-400 7670489

[B34] Kos CH Le TC Sinha S Henderson JM Kim SH Sugimoto H Kalluri R Gerszten RE Pollak MR Mice deficient in alpha-actinin-4 have severe glomerular disease J Clin Invest 2003 111 11 1683 1690 10.1172/JCI17988 12782671 PMC156110

[B35] Shih NY Li J Karpitskii V Nguyen A Dustin ML Kanagawa O Miner JH Shaw AS Congenital nephrotic syndrome in mice lacking CD2-associated protein Science 1999 286 5438 312 315 10.1126/science.286.5438.312 10514378

[B36] Mollet G Ratelade J Boyer O Muda AO Morisset L Lavin TA Kitzis D Dallman MJ Bugeon L Hubner N Podocin inactivation in mature kidneys causes focal segmental glomerulosclerosis and nephrotic syndrome J Am Soc Nephrol 2009 20 10 2181 2189 10.1681/ASN.2009040379 19713307 PMC2754108

[B37] Sellier-Leclerc AL Duval A Riveron S Macher MA Deschenes G Loirat C Verpont MC Peuchmaur M Ronco P Monteiro RC A humanized mouse model of idiopathic nephrotic syndrome suggests a pathogenic role for immature cells J Am Soc Nephrol 2007 18 10 2732 2739 10.1681/ASN.2006121346 17855645

[B38] Bertani T Poggi A Pozzoni R Delaini F Sacchi G Thoua Y Mecca G Remuzzi G Donati MB Adriamycin-induced nephrotic syndrome in rats: sequence of pathologic events Lab Invest 1982 46 1 16 23 6172662

[B39] Lenderink AM Liegel K Ljubanovic D Coleman KE Gilkeson GS Holers VM Thurman JM The alternative pathway of complement is activated in the glomeuli and tubulointerstitium of mice with adriamycin nephropathy Am J Physiol Renal Physiol 2007 293 2 F555 564 10.1152/ajprenal.00403.2006 17522263

[B40] Zheng Z Schmidt-Ott KM Chua S Foster KA Frankel RZ Pavlidis P Barasch J D'Agati VD Gharavi AG A Mendelian locus on chromosome 16 determines susceptibility to doxorubicin nephropathy in the mouse Proc Natl Acad Sci USA 2005 102 7 2502 2507 10.1073/pnas.0409786102 15699352 PMC549022

[B41] Cheng H Fan X Guan Y Moeckel GW Zent R Harris RC Distinct roles for basal and induced COX-2 in podocyte injury J Am Soc Nephrol 2009 20 9 1953 1962 10.1681/ASN.2009010039 19643929 PMC2736764

[B42] Vielhauer V Berning E Eis V Kretzler M Segerer S Strutz F Horuk R Grone HJ Schlondorff D Anders HJ CCR1 blockade reduces interstitial inflammation and fibrosis in mice with glomerulosclerosis and nephrotic syndrome Kidney Int 2004 66 6 2264 2278 10.1111/j.1523-1755.2004.66038.x 15569315

[B43] Hahn H Park YS Ha IS Cheong HI Choi Y Age-related differences in adriamycin-induced nephropathy Pediatr Nephrol 2004 19 7 761 766 10.1007/s00467-004-1487-z 15138873

[B44] Artunc F Nasir O Amann K Boini KM Haring HU Risler T Lang F Serum- and glucocorticoid-inducible kinase 1 in doxorubicin-induced nephrotic syndrome Am J Physiol Renal Physiol 2008 295 6 F1624 1634 10.1152/ajprenal.00032.2008 18768591

